# *Akkermansia muciniphila* - impact on the cardiovascular risk, the intestine inflammation and obesity

**DOI:** 10.3389/abp.2024.13550

**Published:** 2024-11-14

**Authors:** Krzysztof Gofron, Adam Berezowski, Maksymilian Gofron, Małgorzata Borówka, Michał Dziedzic, Wojciech Kazimierczak, Maciej Kwiatkowski, Maria Gofron, Zuzanna Nowaczyk, Sylwia Małgorzewicz

**Affiliations:** ^1^ Students’ Circle of Clinical Nutrition, Medical University of Gdańsk, Gdańsk, Poland; ^2^ Department of Urology and Kidney Transplantation, Nikolay Pirogov Provincial Specialist Hospital, Łódź, Poland; ^3^ Urology Department, Municipal Teaching Hospital in Częstochowa, Częstochowa, Poland; ^4^ Department of Otolaryngology, Laryngological Oncology, Audiology and Phoniatrics, Medical University of Łódź, Łódź, Poland; ^5^ Department of Orthopedics and Traumatology, Medical University of Warsaw, Warszawa, Poland; ^6^ Department of Clinical Nutrition, Medical University of Gdańsk, Gdańsk, Poland

**Keywords:** *Akkermansia muciniphila*, cardiovascular diseases, inflammation, obesity, microbiota

## Abstract

Contemporary scientific discussions are increasingly focusing on *Akkermansia muciniphila* due to its complex influence on intestinal physiology. This article provides a comprehensive analysis of the various effects *Akkermansia muciniphila* has on intestinal inflammation, while also exploring its potential associations with obesity and cardiovascular diseases. A systematic literature search was conducted using PubMed, Google Scholar, and ResearchGate with the following keywords: *Akkermansia muciniphila*, obesity, cardiovascular risk, and inflammatory bowel diseases. The aim of our mini-review was to examine the impact of *Akkermansia* bacteria on the intestines, cardiovascular system, and its relationship with obesity. Through a detailed review of current literature, the article seeks to elucidate the complex interactions of *Akkermansia muciniphila* within the human body, highlighting its potential contributions to health improvement and medical interventions. Research indicates that *Akkermansia muciniphila* positively correlates with maintaining intestinal health, modulating the cardiovascular system, and aiding in weight management. However, the number of studies available is small, and the effects of *Akkermansia muciniphila* on human health require further research.

## Introduction

**FIGURE 1:**
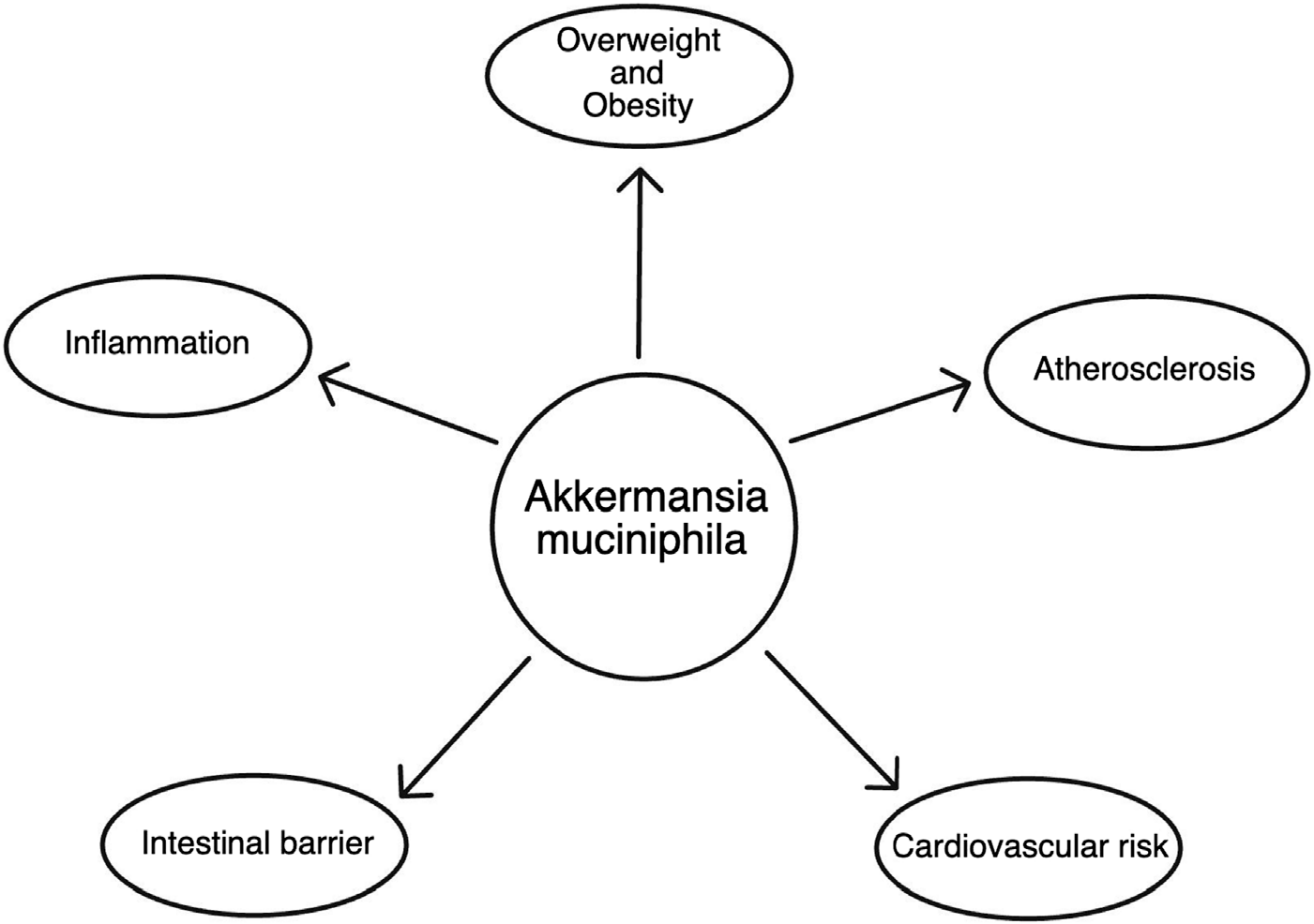
The scheme of *Akkermansia muciniphila* impact on health.

In recent years, the microbiome has emerged as a key player in influencing various aspects of human health. Among the myriad of microorganisms inhabiting the human gut, *Akkermansia muciniphila*, isolated from feces and named in 2004 by a group of Dutch scientists, led by professor Willem M. de Vos and PhD. Muriel Derrien has gained considerable attention for its potential role in shaping our overall wellbeing. This microscopic bacterium is particularly intriguing due to its distinctive ability to thrive on mucin, a key component of the protective mucus layer lining the intestines. As research continues to unveil the intricate relationship between *Akkermansia muciniphila* and human health, its profound impact on inflammation within the gastrointestinal tract and the broader implications for cardiovascular health have become subjects of intense scientific exploration (Derrien et al., 2010). This article delves into the multifaceted influence of *Akkermansia muciniphila* on the inflammatory processes within the intestines and its consequential effects on the obesity and cardiovascular system (Figure 1).

Due to the frequent occurrence of diseases such as obesity and cardiovascular diseases, it is important to prevent them through various options, as well as to support available treatments. Modulation of the gut microbiome may be one of the potential possibilities. Therefore, the aim of our mini review was to examine the impact of *Akkermansia* bacteria on the effects on the intestines, cardiovascular system and the relationship with the obesity.

## Methods

A systematic literature search was conducted using the PubMed, Google Scholar and ResearchGate with the following keywords: *Akkermansia muciniphila*, obesity, cardiovascular risk, inflammatory bowel diseases. We found 24 trials included studies on humans and animals in 2007–2023 years regarding *A. muciniphila’s* impact on the intestines, cardiovascular risk and obesity. 11 of them were rejected because they did not meet the criteria for an observational or interventional study.

### Characteristics of *Akkermansia muciniphila*

*A. muciniphila* is an anaerobic, gram-negative rod, representing the sole bacterium from the Verrucomicrobiales family in the human body (Derrien et al., 2010). It colonizes the mucosal layer of the crypts in the colon and rectum. Devoid of spore-forming capabilities and independent motility, this bacterium specializes in the degradation of mucins - glycoprotein conjugates produced by epithelial cells to ensure their proper functioning. Elevated production of these proteins, integral to mucosal integrity, correlates with an increased likelihood of colorectal cancer, particularly involving the MUC-1 protein (Nath and Mukherjee, 2014).

*A. muciniphila* makes its presence in the human gut as early as the first year of life, acquired from maternal milk, constituting approximately 1%–3% of the bacterial population (Collado et al., 2007). Its levels are diminished in individuals with overweight or obesity, and they exhibit an inverse correlation with age (Collado et al., 2007; Dao et al., 2016; Dao et al., 2019). Preliminary studies conducted on a group of nine individuals during Ramadan, simulating intermittent fasting conditions, unequivocally indicate a significant rise in the quantity of *A. muciniphila* in the gut during intermittent fasting (Dao et al., 2016; Dao et al., 2019; Mousavi et al., 2022). Crucially, research on a cohort of patients who underwent bariatric surgery did not confirm a similar increase in *A. muciniphila* levels despite the requisite dietary changes, suggesting that the surge in *A. muciniphila* is specific to intermittent fasting rather than a dietary shift (Dao et al., 2016; Dao et al., 2019). However, further studies on a larger sample size are essential for validation. Although there is no evidence supporting the influence of diet on *A. muciniphila* levels, other factors come into play such as metformin, a first-line medication for type 2 diabetes, which has been found to statistically positively correlate with *A. muciniphila* levels in the gut (Shin et al., 2014).

*A. muciniphila* is categorized into four phenotypic groups, AmI to AmIV (Becken et al., 2021). The most extensively studied strain and widely available as a probiotic is the pasteurized MucT strain, belonging to the AmI group. Despite initial assumptions of being strictly anaerobic, *A. muciniphila* has demonstrated adaptability to aerobic environments. Even low oxygen concentrations can promote its growth, as evidenced by studies on various strains of the bacterium. Strains belonging to AmII exhibit relatively good tolerance to aerobic conditions, with a survival rate of 60% after 24 h, whereas AmIV strains display very low survival at 0.01% under the same conditions after only 12 h (Becken et al., 2021). *A. muciniphila* also participates in the transformation of cobalamin precursors (Cbi) into cobalamin (vitamin B12), although it cannot synthesize it *de novo* (Mok et al., 2020). To date, there is no evidence supporting the possibility of human infection by *A. muciniphila* with research indicating the absence of negative effects associated with the administration of this bacteria (Depommier et al., 2019).

### Cardiovascular impact of *Akkermansia muciniphila*

*Akkermansia muciniphila* significantly reduces local inflammation of the vascular endothelium, leading to atherosclerosis. An approximately 2.4-fold increase in the quantity of *A. muciniphila* results in a 72.59% decrease in C-reactive protein (CRP) levels, measured by a highly sensitive method (Shin et al., 2014). Studies on mice in 2016 demonstrated that in mice fed a high-fat diet, administering live cultures of this bacterium reduces the adhesion of macrophages, crucial in atherosclerotic plaque formation to the endothelium by inhibiting the expression of tumor necrosis factor-alpha (TNF-alpha), MCP-1, and ICAM-1 (Li et al., 2016; Qu et al., 2021). Additionally, *A. muciniphila* significantly lowers soluble TNF receptor II (sTNFR II), reducing the severity of atherosclerosis. Importantly, the mechanism influencing atherosclerosis does not involve lowering lipid and sugar levels in circulating blood, nor does it affect the concentration of adiponectin, an anti-inflammatory substance (Li et al., 2016; Zhong et al., 2022). *A. muciniphila* plays a crucial role in weight control by reducing the absorption of lipids, achieved through maintaining the proper thickness of the intestinal mucosal layer, made possible by this bacterium’s ability to adhere to the mucous membrane (Reunanen et al., 2015). These findings were observed exclusively in mice fed a high-fat diet, with studies on mice fed a normal diet showing no significant impact on these processes (Li et al., 2016).

Another significant cardioprotective action of *A. muciniphila* is counteracting arterial calcification, a component of atherosclerotic disease. This process involves the deposition of calcium salts in atherosclerotic plaques, leading to increased stiffness and, among other things, arterial hypertension. Short-chain fatty acids, propionate, and butyrate produced by human gut bacteria largely contribute to arterial calcification. Propionate reduces arterial calcification, while butyrate increases the intensity of this process (Zhong et al., 2022). Administering live *A. muciniphila* induces the production of, among other things, propionate, which has a protective effect on arterial calcification. No positive effects on the production of short-chain fatty acids were observed when pasteurized bacteria were administered (Yan et al., 2022).

Atrial fibrillation is the most common arrhythmic pathology in society, characterized by asynchronous atrial contractions often exceeding 150 beats per minute. This leads to blood stasis in these areas of the heart, which can result in clot formation and, ultimately, cause embolisms, with the most dangerous being those reaching the brain, often leading to a stroke. Atrial fibrillation is induced by factors such as cardiomyopathies, thyroid disorders, lung diseases, organ obesity, and less obvious factors, including a decrease in ambient temperature (Fustinoni et al., 2013). The latter also has a significant impact on the gut microbiota, including *A. muciniphila*, whose quantity is reduced at lower temperatures. It is *A. muciniphila* that exerts a significant influence on the frequency and duration of atrial fibrillation occurrences (Luo et al., 2022). Administering *A. muciniphila* cultures reduces the synthesis of trimethylamine (TMA), which is transformed into Trimethylamine N-oxide (TMAO). TMAO is responsible for recruiting M1 macrophages, which, by increasing inflammation associated with cytokine production, induce pyroptosis of cardiomyocytes in the heart atria (Luo et al., 2022). This relationship did not occur after administering pasteurized bacteria.

*A. muciniphila* demonstrates significant therapeutic value on a potentially large scale in the treatment of cardiovascular diseases. However, all existing studies clearly indicate that administering live bacteria is necessary to achieve a therapeutic effect. The impact on the aforementioned processes suggests the greatest potential application in Western diets, which largely rely on low-nutrient value products with high-saturated fat levels. The Western diet often leads to weight gain, resulting in overweight or even obesity and is the cause of systemic inflammation (Malesza et al., 2021; Warmbrunn et al., 2024). Both obesity and overweight contribute to the development of up to 50 different diseases, with a particular focus on the cardiovascular system. The application of live cultures of *A. muciniphila* reduces pyroptosis of cardiomyocytes, counteracting the development of atrial fibrillation, thereby reducing the risk of strokes (Christ et al., 2019). Moreover, *A. muciniphila* reduces the risk of ischemia heart disease in the African-Surinamese group playing a crucial role in cardiovascular diseases (Christ et al., 2019). Additionally, an increased abundance of this bacterium reduces endothelial inflammation, preventing the formation of atherosclerotic plaques and decreasing calcifications in already existing plaques. The reduction in both the quantity and hardness of atherosclerotic plaques contributes to a lower risk of unstable angina. Weight reduction, facilitated by *A. muciniphila*, also lowers average blood pressure, ultimately relieving the workload on the cardiac muscle counteracting the hypertrophy of the left ventricle and the development of aortic aneurysms. Further research involving a larger sample size is essential for the application of potential therapy. Main studies dealing with the effects of *A. muciniphila* in cardiovascular diseases are presented in Table 1.

**TABLE 1:** Main studies dealing with the effects of *Akkermansia muciniphila* in cardiovascular diseases.

Study	Description	Design	Size	Patients	Length	Outcomes
Li et al. (2016)	Healthy	Animal study	—	Apoe^−/−^ mice	8 weeks	*A. muciniphila* prevented inflammation induced by a Western diet both in the circulation and in local atherosclerotic plaques
Yan et al. (2022)	Healthy	Observational cohort study	92	Adults	—	*A. muciniphila* abundance negatively correlates with vascular calcification
Luo et al. (2022)	Healthy	Animal study	7	Rats	3 weeks	Oral supplementation of *A. muciniphila* mitigated pro-atrial fibrillation properties induced by cold exposure
Warmbrunn et al. (2024)	Healthy	Prospective cohort study	3,860	Adults	—	*A. Muciniphila* abundance is protective against ischemia heart disease

### Impact of *Akkermansia muciniphila* on the intestine

The main mechanism of action of *A. muciniphila* in the body is its involvement in the formation of the intestinal mucosal layer, where it resides. A healthy mucosal layer is responsible, among other things, for the efficient absorption of nutrients from food and the human immune response. A deficiency of *A. muciniphila* in the gut microbiota can lead to severe pathological conditions, such as inflammatory bowel diseases, a compromised host immune response, invasive microorganisms, or increased susceptibility to the detrimental effects of toxins on the body.

Inflammatory bowel disease is a term used to describe a group of chronic intestinal inflammations. The two main types are Crohn’s disease (CD) and ulcerative colitis (UC). Despite many similarities, these diseases differ in several aspects. CD can affect any part of the digestive system, while UC primarily affects the large intestine. Another difference lies in the appearance of intestinal inflammation; CD creates skip lesions, whereas UC inflammation spreads continuously. It has been demonstrated that *A. muciniphila* supplementation significantly reduces symptoms of dextran sulfate-induced acute colitis and that its quantity is reduced in inflammatory bowel disease (Qu et al., 2021). It is crucial to minimize intestinal inflammation as much as possible, as inflammatory bowel diseases significantly increase the likelihood of developing colorectal cancer (Yao et al., 2019), (see Table 2).

**TABLE 2:** The impact of the *Akkermansi*a *muciniphila* on intestines.

Study	Disease	Design	Size	Patients	Length	Outcomes
Qu et al. (2021)	Healthy	Animal study	10	Dextran Sulfate Sodium - fed mice	18 days	Oral administration of *A. muciniphila* significantly ameliorated the symptoms in dextran sulfate sodium (DSS)-induced acute colitis
Collado et al. (2007)	Healthy	Observatory study	249	Infants and Adults	—	*A. muciniphila* is correlated with normal mucosa development
Liu et al. (2022)	Healthy	Animal study	30	Dextran Sulfate Sodium - fed mice	2 weeks	*A. muciniphila*-based mechanisms play a fundamental role in driving the divergent induction of suppressive RORγt+ Treg cells in the gut-specific microenvironment

The first mechanism through which chronic inflammatory bowel symptoms are alleviated (reducing the severity of intestinal inflammation, i.e., depth and extent at the histological level, as well as mitigating weight loss associated with reduced nutrient absorption) is the activation of NLRP3, which exhibits anti-inflammatory and immune actions (Qu et al., 2021; Guo et al., 2020). NLRP3 influences the production of IL-18, which plays a crucial role in creating the protective barrier of the intestine (Guo et al., 2020; Chiang et al., 2022). IL-18 regulates the gut microbiota, reducing the development of invasive colonies. Studies on mice showed significantly reduced mucin production from goblet cells in mice with decreased IL-18 levels compared to mice with normal levels (Chiang et al., 2022), demonstrating a positive correlation between IL-18 and mucin levels, whose breakdown fulfills the carbon and nitrogen needs of *A. muciniphila* (Derrien et al., 2010; Collado et al., 2007). Notably, there is an increase in the levels of Muc-2 and Muc-3 proteins.

Another mechanism influenced by *A. muciniphila* is the activation of TLR4 receptors, whose levels are increased in patients with both Crohn’s disease and ulcerative colitis (Liu et al., 2022; Hayashi and Nakase, 2022). The positive function of TLR4 in the body is the recognition of PAMPs and DAMPs, responsible for the human immune response in practice, while negatively influencing an increased likelihood of developing inflammatory bowel diseases (Liu et al., 2022; Moresco et al., 2011). The TLR-4-dependent anti-inflammatory response mainly depends on RORγt+ regulatory T lymphocytes, whose levels are reduced in the intestine during inflammatory bowel diseases (Liu et al., 2022). *A. muciniphila* can also activate TLR-2, but about a tenfold increase in the number of bacteria is required for this, and the activation of this receptor does not significantly impact the immune response stimulated by TLR receptors (Becken et al., 2021). TLR-2 and TLR-4 receptors are much more activated by phenotypic groups AmII and AmIV than by the AmI group, to which the MucT strain belongs.

An additional protective effect of *A. muciniphila* on the intestine is the inhibition of the expression of TNF-alpha, MCP-1, and ICAM-1, which, similar to the vascular endothelium, acts anti-inflammatory and inhibits the action of macrophages.

### The association with obesity

Obesity and overweight are among the primary lifestyle diseases, affecting nearly one-third of the global population every day. Both disorders lead to various pathological conditions, predisposing individuals to diseases in almost every system, including the cardiovascular, musculoskeletal, respiratory, hormonal, and others. Cardiovascular diseases, responsible for approximately half of global deaths, prove to be particularly deadly. Although metabolic diseases result from various factors, such as genetic predispositions, gut microbiome, hormonal system, diet, upbringing during childhood, and the culture in which a person was raised, the most significant impact on these disorders still comes from a person’s diet. The hope for treatment lies in many therapies, both non-pharmacological and pharmacological, but the key to success is their simultaneous application (Cao et al., 2023).

Therapy typically begins with non-pharmacological treatment focusing on diet modification and increased physical activity. This is the healthiest way to reduce body weight but is often ineffective due to individuals’ habits and accompanying health conditions that limit their ability to move.

When non-pharmacological treatment proves ineffective, the next steps involve introducing medications to assist in weight loss. These medications are usually chosen with attention to coexisting diseases. Examples include glucagon-like peptide-1 (GLP-1) analogs used in type II diabetes patients or thyroxine (T4) analogs in the case of hypothyroidism both proving to reduce weight while treating the underlying cause.

Let’s focus on pharmacological treatment by influencing the gut microbiome. A promising and therapeutically valuable approach is the therapy using *A. muciniphila* bacteria. A widely available probiotic containing pasteurized *A. muciniphila* bacteria, specifically the MucT strain of the AmI group, has emerged worldwide. Oral therapy with *A. muciniphila* significantly reduces serum glucose and triglyceride levels, as well as tissue insulin resistance (Dao et al., 2016; Depommier et al., 2019). The reduction in serum glucose and triglyceride levels is likely associated with improved liver function, as indicated by decreases in aspartate aminotransferase (AST) and gamma-glutamyl transferase (GGT) levels, used as markers of liver dysfunction (Dao et al., 2016; Xu et al., 2023). An increase in *A. muciniphila* levels in patients is linked to a decrease in the average volume of adipocytes, making these individuals metabolically healthier (Dao et al., 2016). The adoption of a healthy diet by patients promotes and facilitates weight loss in individuals with higher levels of *A. muciniphila* (Dao et al., 2016; Cao et al., 2023). Results indicating no loss of muscle mass in patients who lost weight and had a higher quantity of *A. muciniphila* compared to those with a lower quantity of this bacterium prove to be very valuable. The studies also demonstrated the absence of the rebound effect in patients (Cao et al., 2023). The reduction in insulin resistance due to A. muciniphila lowers the risk of developing type 2 diabetes and reduces drowsiness and fatigue. Additionally, it reduces hunger attacks leading to weight gain. It also decreases the activity of dipeptidyl peptidase-IV (DPP-IV) (Depommier et al., 2019), which is responsible for the breakdown of glucagon-like peptide 1 (GLP-1), responsible for inhibiting hunger and intensifying the feeling of fullness. Due to this many positive effects it leads to the assumption that therapies based on *A. Muciniphila* may become world spread. The studied focused on association between *A. muciniphila* and obesity are presented in Table 3.

**TABLE 3:** Main studies dealing with the effects of *Akkermansia muciniphila* in bodyweight management.

Study	Disease	Design	Size	Patients	Length	Outcomes
Dao et al. (2019)	Obesity	Non-randomised prospective study	65	Adults	12 months	*A. muciniphila* abundance does not affect glucose tolerance and insulin sensitivity in patients after gastric bypass surgery
Shin NR (2014)	Healthy	Animal study	24	HFD-fed mice	6 weeks	*A. muciniphila* administration reduse adipose tissue inflammation
Depommier et al. (2019)	Overweight and obesity	Randomised double blind study	32	Adults	3 months	Increased *A. muciniphila* abundance negatively correlates with insulin resistance, inflammation markers and liver disfunction markers
Dao (2016)	Overweight and obesity	Randomised control study	49	Adults	3 months	Increased *A. muciniphila* abundance positively correlates with insulin sensitivity
Cao et al. (2023)	Obesity	Randomised double blind study	37	Adults	4 months	Increased *A. muciniphila* abundance positively correlates with weight loss

## Discussion and conclusion

The continuous rise in diseases related to overweight and obesity, including cardiovascular diseases, highlights the need for an effective and safe method to address this issue. Diseases associated with excess body weight are closely linked to a systemic inflammatory state triggered by adipocytes and by an imbalance in gut microbiota. It should be noted that while inflammatory bowel diseases (IBD) and metabolic diseases may seem etiologically distinct, treatment for IBD may increase the risk of metabolic conditions, including obesity. Furthermore, inflammation is a common factor in both, heightening the risk of further complications. *Akkermansia muciniphila* meets the criteria for potential treatment, as there is no evidence of human infection by this bacterium, and its use has shown promising results in promoting safe and sustainable weight loss, alongside a reduction in systemic inflammation. There are several reasons to believe that *A. muciniphila* therapy could have a positive impact on cardiovascular diseases, particularly atherosclerosis, which is linked to endothelial inflammation. Additionally, increasing the levels of *A. muciniphila* in the gut appears to alleviate the negative symptoms of chronic inflammatory bowel diseases, such as ulcerative colitis (UC) and Crohn’s disease (CD).

*Akkermansia muciniphila* has demonstrated potential for comprehensive treatment of these diseases. Given the rising global prevalence of both inflammatory bowel diseases and metabolic disorders, this is a particularly important issue. Moreover, treatment with *Akkermansia muciniphila* is relatively affordable. However, the number of studies exploring the association between *A. muciniphila* and obesity, cardiovascular risk, and intestinal health is limited, and further research is needed to fully understand its effects on human health.

## Future direction

The fundamental issue that needs to be addressed is the assessment of the safety of consuming live cultures of *Akkermansia muciniphila*. So far, research suggests a much broader potential for *A. muciniphila* in this form to achieve positive cardiovascular effects. It is essential to direct further research towards the circulatory system because, despite many indications of its positive impact, there is still a lack of large-scale studies involving a greater number of individuals to justify the implementation of such therapy.
